# Development of a support system for creating disaster prevention maps focusing on road networks and hazardous elements

**DOI:** 10.1186/s42492-021-00089-7

**Published:** 2021-08-19

**Authors:** Kaname Takenouchi, Ikuro Choh

**Affiliations:** 1 Graduate School and Department of Intermedia Art and Science, Waseda University, 3-4-1 Okubo, Shinjuku-ku, Tokyo, Japan; 2 Waseda University, 3-4-1 Okubo, Shinjuku-ku, Tokyo, Japan

**Keywords:** Disaster prevention map, Road network analysis, Hazardous elements, Simulation of evacuation drill

## Abstract

As a disaster prevention measure based on self-assistance and mutual assistance, disaster prevention maps are being created with citizen participation throughout Japan. The process of creating disaster prevention maps is itself a disaster prevention measure that contributes to raising awareness of disaster prevention by promoting exchange and cooperation within the region. By focusing on relations between road networks and hazardous elements, we developed a system to support disaster prevention map creation that visualizes roads at high risk during a disaster and facilitates the study of evacuation simulations. This system leads to a completed disaster prevention map in three phases. In the first phase, we use a device with GPS logging functions to collect information related to hazardous elements. In the second phase, we use Google Maps (“online map,” below) to visualize roads with high evacuation risk. In the final phase, we perform a regional evaluation through simulations of disaster-time evacuations. In experimental verifications, by conducting usability tests after creating a disaster prevention map in the target area, we evaluated the system in terms of simple operability and visibility. We found that by implementing this series of processes, even users lacking specialized knowledge regarding disaster prevention can intuitively discover evacuation routes while considering the relations between visualized road networks and hazardous elements. These results show that compared with disaster prevention maps having simple site notations using existing WebGIS systems, disaster prevention maps created by residents while inspecting the target area raise awareness of risks present in the immediate vicinity even in normal times and are an effective support system for prompt disaster prevention measures and evacuation drills.

## Introduction

After the Great East Japan Earthquake struck on March 11, 2011, the government agencies that were supposed to provide support to disaster victims became disaster victims themselves, and their administrative functions were paralyzed. This situation demonstrated the limitations of public assistance in large-scale, wide-area disasters. Even so, it also demonstrated that cooperation through self-assistance, mutual assistance, and public assistance are indispensable aspects of disaster countermeasures.

Regarding disaster prevention measures in Japan, lessons learned from large earthquakes have reaffirmed the importance of self-assistance to protect one’s own life as well as mutual assistance through cooperation with others in the local community. For self-assistance and mutual assistance efforts, it is essential to prepare for large-scale disasters before they occur. It is therefore desirable to utilize hazard maps and disaster prevention maps issued by local governments to determine what disaster prevention measures can be taken in advance. As stipulated by the Earthquake Disaster Countermeasures Ordinance, the Tokyo Metropolitan Government conducts a regional earthquake risk measurement survey every five years and publishes regional risk measures based on the results. Regional risk levels are calculated for 5,177 districts and streets in metropolitan Tokyo, including building risks, fire risks, and the difficulty of carrying out activities in the midst of a disaster [1].

However, the storage and utilization rates of these hazard maps are low, and thus they tend to not to be used as disaster prevention tools [2]. One possible cause for this is that the information provided is not linked to residents’ evacuation behavior.

In contrast, workshops for creating disaster prevention maps are being held throughout Japan for the purpose of raising awareness about disaster prevention and mitigating damage that results from disasters. It has been confirmed that the creation of disaster prevention maps contributes to raising awareness of disaster prevention among participants [3], and it is expected that the disaster prevention capabilities of self-assistance and mutual assistance will be improved. Against this background, various studies have supported the creation of disaster prevention maps, particularly through use of WebGIS [4–7].

Enokida et al. [4] developed a system for WebGIS-based recordings of disaster prevention information collected while walking through towns, demonstrating the system’s usefulness through comparative verifications with the creation of conventional paper-based disaster prevention maps. Tajima et al. [5] developed a support system for smartphone-based disaster prevention map creation and evaluated the storage and convenience of disaster prevention information linked with GPS. In these systems, the process of collecting disaster prevention information while walking through towns and experiencing the task of storing information in WebGIS produced results that contributed to raising participants’ interest in disaster prevention and their awareness of disaster prevention.

Murakoshi et al. [6] designed a system that integrates WebGIS and social media and evaluated its operation. The developed system shares disaster information on a map in normal times, and it has contributed to raising awareness of disaster prevention among local residents.

Kubota et al. [7] developed a participation-type GIS and evaluated its operation. By providing point rewards as compensation for information, they devised a way to incentivize user participation in the system. Specifically, the system awards points for providing any local information, not just that related to disaster prevention, and these points can be redeemed at local events and the like.

Many of these systems aim to collect information necessary for disaster prevention (dangerous locations, evacuation sites, and so on) on a WebGIS map. However, the role of a disaster prevention map is not only to disseminate disaster images and disaster information, but also to consider risk aversion in disasters that may cause damage in the future. In particular, considering risk avoidance in disasters requires identification of roads with higher risk during evacuations than in normal times. In other words, considering hazardous elements in the areas we live in that might lead to secondary damage during a disaster and situations in which dangers have already emerged, as well as understanding the characteristics of local road networks, is an effective way to protect ourselves during a disaster.

The space syntax theory proposed by Hillier et al. [8] is a method for analyzing the characteristics of road networks. This theory quantifies connections between intersections (nodes) and roads (edges), which are the main elements of a road network. Because it can analyze road characteristics, it is used in various studies as a method for analyzing spatial structures in cities. In particular, the following studies have clarified relations between phenomena occurring in regions and road networks. A study by Takahashi et al. [9] analyzed relations between the physical quantities of greenery perceived by humans in areas and road network conditions (Int.V, an index indicating the extent of road connectivity; omitted below) in order to demonstrate local trends in regional greenery. A study by Nagaie et al. [10] revealed trends in local crime risk by analyzing relations between the occurrence of street crime, crime risk as recognized by the police, and road network conditions. These studies are characterized by their clarification of regional characteristics through the quantitative analysis of latent factors in the city.

We considered whether the methods used in these previous studies, which analyzed spatial structures by focusing on specific road network factors, might be applied to the issue of regional disaster prevention that is addressed in the present study. To that end, we focused on the relations between road network conditions and hazardous elements in order to develop a system based on a space syntax theory approach, which allows for investigation of disaster evacuation simulations by visualizing roads with high evacuation risk.

In the remainder of this paper, we first show the process of creating a disaster prevention map using this system, then we outline the space syntax theory used in each phase, define the hazardous elements, and present the device specifications and an overview of the system. We then present an analysis and its results based on a verification of the disaster prevention map created using this system for the three target areas. From these results, we consider the effectiveness of the proposed system as a means of support for disaster prevention measures and evacuation training.

## Methods

The support system for creating disaster prevention maps proposed in this study is characterized by its use of a series of processes (Table 1) that can be carried out even by people who do not have specialized knowledge about disaster prevention, meaning that disaster prevention maps can be created through the cooperation of individuals or groups. In particular, we considered the device design and the online map display at each process phase so that users creating disaster prevention maps can complete them without having to perform complicated operations.

**Table 1:** Process of making disaster prevention map

Phase 1	Collection and evaluation of hazardous elements using device
	(GPS loger).
Phase 2	Visualization and analysis of roads with high evacuation
	risk focusing on the relationship between road networks
	and hazardous elements.
Phase 3	Simulation of evacuation routes at the time of disaster.

### Space syntax theory

The support system for creating disaster prevention maps proposed in this study uses space syntax theory as a method for analyzing the characteristics of road networks. This theory, which was proposed by Hillier [6] of the University of London in the 1970s, analyzes spatial connections. This theory has been used in research, mainly to analyze spatial structures in architectural interiors and urban spaces. Axial analysis is a method used in space syntax theory to segment indoor and outdoor spaces and calculate a numeric value (an integration value) representing spatial connectivity based on relations between intersections (nodes) and roads (edges). In other words, space syntax theory can reveal seemingly complex patterns in indoor and outdoor spaces as structures that follow rational rules.

Axial analysis calculates an integration value (“Int.V”) as an index of spatial connectivity. The following describes the calculation of Int.V using axial analysis.

**Fig. 1:**
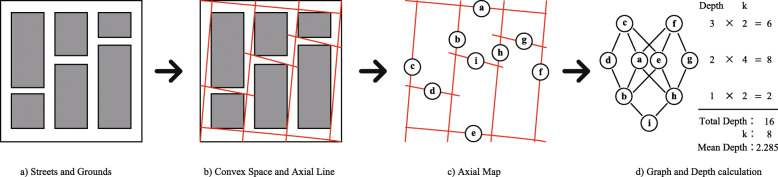
Relationship between Convex Space and A-Line. **a)** Streets and Grounds. **b)** Convex Space and Axial Line. **c)** Axial Map. **d)** Graph and Depth calculation

In axial analysis, the analyzed space is segmented into spatial units called convex spaces. Next, using isovists (regions visible from a given point in space) as hints, we draw axial lines (“A-lines”) that segment the convex space to the greatest extent and with the fewest A-lines possible. From this, we can represent the connectivity between the convex space and the A-lines as a graph (Fig. 1). We can represent the mean depth (MD) and the total depth (TD) as

1 $$\begin{array}{@{}rcl@{}} &&MD_{i} = \frac{TD_{i}}{k-1}  \\ &&TD_{i}=\sum^{k}_{j=i}Depth_{ij} \end{array} $$

Next, the degree of depth (*RA*_*i*_) for all paths of a given A-line can be calculated as

2 $$\begin{array}{@{}rcl@{}} &&RA_{i} = \frac{2(MD_{i}-1)}{k-2} \end{array} $$

where *k* is the total number of vertices. The *RA*_*i*_ value depends on the analyzed A-line vertices, and thus can be standardized as *RRA*_*i*_ as

3 $$\begin{array}{@{}rcl@{}} &&RRA_{i} = \frac{(k-1)(k-2)}{2\left[k\left\{{log_{2}\Bigl(\frac{k+2}{3}}\Bigr)\right\}+1\right]} \end{array} $$

Furthermore, by taking the reciprocal of *RRA*_*i*_,*IntV*_*i*_ can be shown as

4 $$\begin{array}{@{}rcl@{}} &&IntV_{i} = \frac{1}{RRA_{i}} \end{array} $$

Because Int.V is the reciprocal of the depth, the higher its value, the shallower the spatial depth and the stronger the connectivity with other spaces. Conversely, lower values indicate greater spatial depth and weaker connectivity. In other words, a given A-line with a high Int.V value indicates a lively space with many pedestrians in the target area.

When all A-lines in a targeted area are considered, the situation is similar to automobile traffic. In this case, Int.V is globally indicated as Int.V-G. However, when the range of the target area is limited, the situation is similar to pedestrian traffic, and this Int.V is locally indicated as Int.V-L. This study targets the evacuation of pedestrians, and thus we use the local application with a radius of 3, allowing us to calculate Int.V in the range of Depth3 (the default value) from the desired vertex. In this study, we visualized road network conditions in targeted areas on online maps, as calculated using space syntax theory. To calculate Int.V, we used depthmapX [11], analysis software based on graph theory developed by Turner [12]. Referencing the Int.V values calculated using depthmapX, we used the Google Maps application programming interface (API) to display road network conditions on an online map, using a color gradation in which warmer colors indicate good road connectivity, and cooler colors indicate bad connectivity. We performed these operations on online maps for each target area where experimental verifications were performed.

### Hazardous elements

**Fig. 2:**
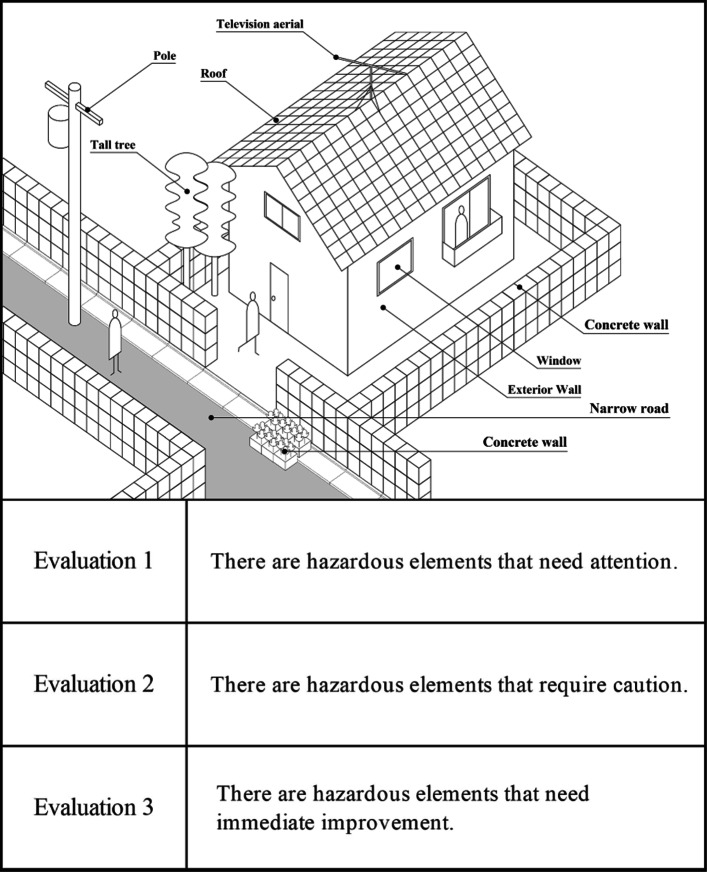
Location of hazardous elements and their evaluation criteria

The 2018 Northern Osaka Earthquake caused a concrete block wall at an elementary school to collapse, killing one student. Based on lessons learned from such accidents, the Ministry of Land, Infrastructure, Transport and Tourism is leading efforts throughout Japan to ensure the safety of concrete block walls and other structures during earthquakes [13]. However, there are innumerable hazards other than walls that can cause secondary damage in the event of a local disaster. Many existing disaster prevention maps outline regional disaster prevention information such as disaster prevention areas, evacuation sites, and evacuation routes, with the aim of reducing damage from natural disasters and applying them to disaster prevention measures, but these do not yet organize factors for secondary damage in the event of a disaster. Therefore, in the disaster prevention map creation proposed in this study, we define elements such as old wooden buildings, concrete block walls, utility poles, and narrow roads as hazardous elements that are likely to cause secondary damage to roads during a disaster, and we define criteria for subjectively evaluating those hazardous elements in three phases (Fig. 2).

### Device

**Fig. 3:**
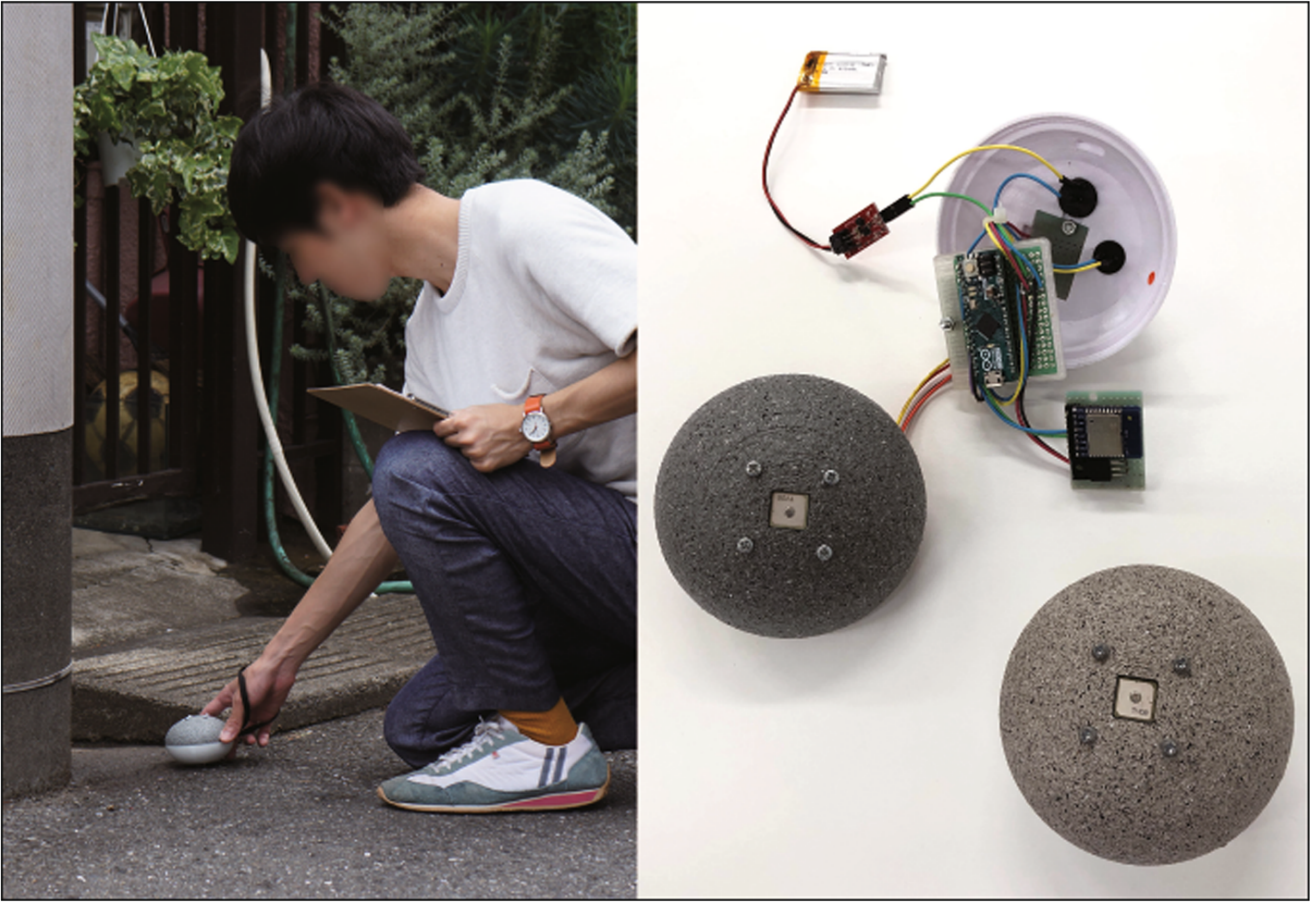
Device for collecting and evaluating hazardous elements

Many existing systems that support the creation of disaster prevention maps include the task of registering disaster prevention information collected using devices such as smartphones in WebGIS, so reflecting disaster prevention information in maps requires users to perform editing tasks. Therefore, in this system we eliminated the need to record disaster prevention information in WebGIS as often seen in existing systems, and instead developed a device equipped with a GPS logger that can reflect the gathered disaster prevention information on an online map in real time (Fig. 3).

This device is characterized by its ability to evaluate hazardous elements through simple operations in three phases, and to reflect location information on an online map in real time. In addition, each device can be manufactured at a low cost. We used Google Maps to link the devices when creating the online map. We adopted Google Maps in consideration of the fact that it is highly versatile in terms of data-sharing and information presentation, and because utilizing its API facilitates rapid development.

**Fig. 4:**
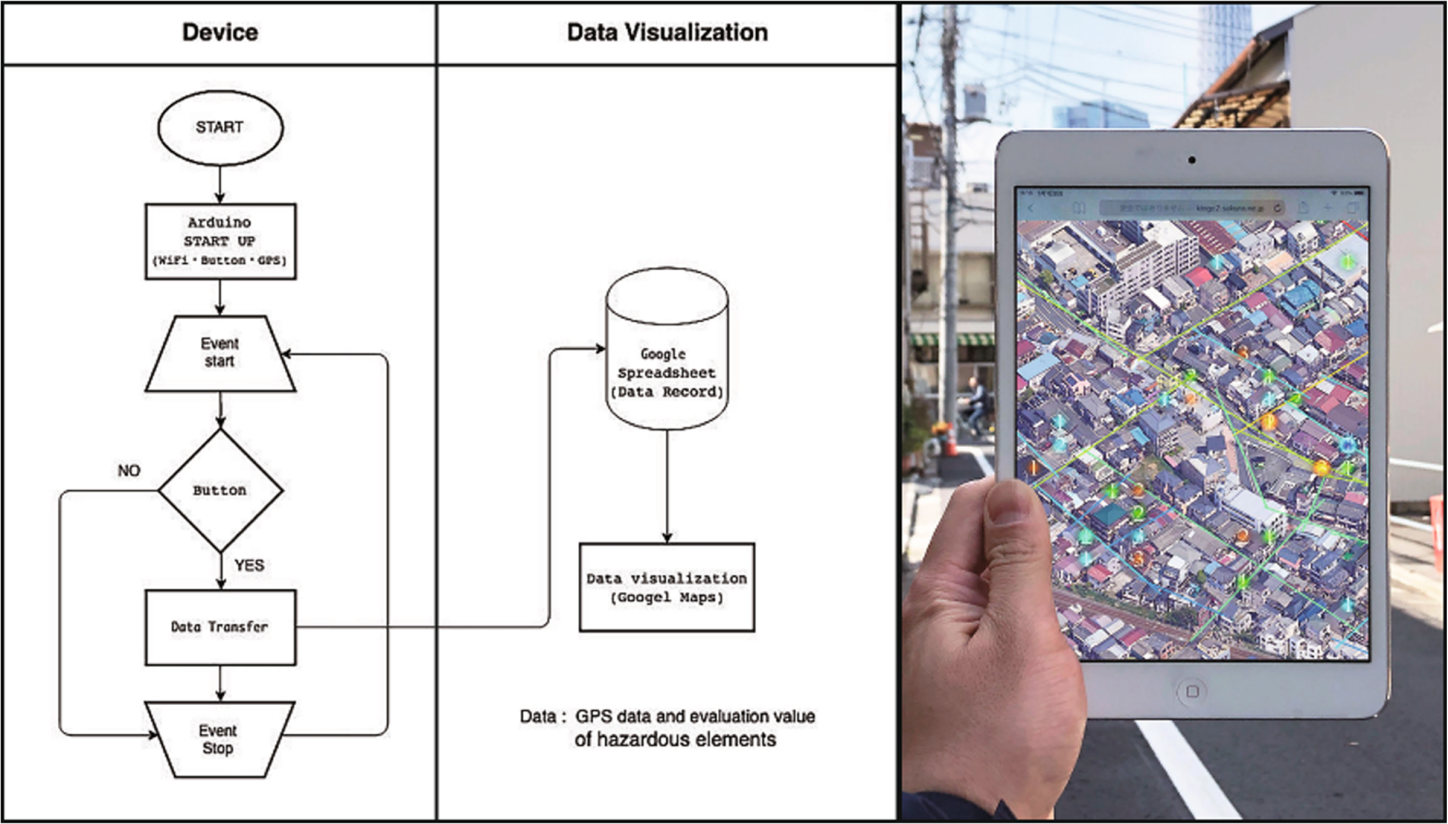
Program flowchart of device

This device was inspired by signposts made of piled stones (cairns), and was therefore designed to resemble a stone. Cairns have long been erected to convey information about a place to others, and thus can be considered a communication tool. In planning disaster prevention, we consider the act of building cairns for sharing disaster prevention information to be an important method for raising awareness of disaster prevention, and thus we developed a device having a GPS logger function for collecting disaster prevention information. By simply placing the device on the ground, location information can be collected, and hazardous elements can be subjectively evaluated in three phases (Fig. 2). The operating environment of the device is an Arduino Micro microcontroller, and the main unit is manufactured using 3D CAD and a 3D printer. Evaluation values for hazardous elements and location data collected by the device are uploaded via a built-in Wi-Fi module and then directly recorded into a Google Sheets spreadsheet using JavaScript. We also used JavaScript to construct a system that can reflects information in Google Sheets in real time (Fig. 4).Hazardous elements displayed on the online map are colored according to the device, and a heat map display function is added.

### Simulation of evacuation route

**Fig. 5:**
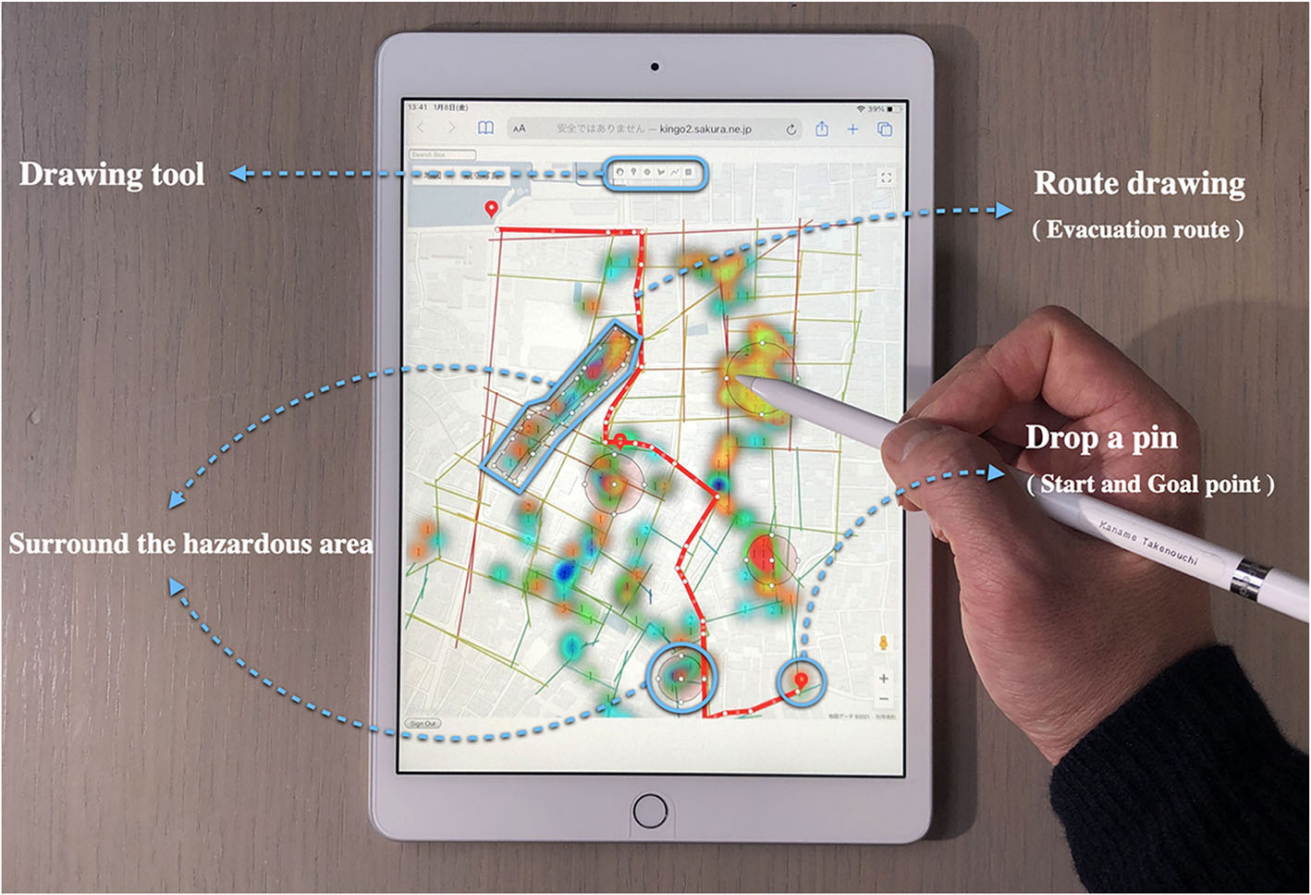
Simulation of evacuation route at time of disaster

The support system for disaster prevention map creation proposed in this study does not aim to organize disaster prevention information collected on a map by walking around town, but rather to complete disaster prevention maps that utilize collected disaster prevention information in order to consider evacuation behaviors for risk avoidance. Accordingly, the proposed system has functions constructed using JavaScript that allow for the recording of effective evacuation routes, evacuation sites, dangerous areas, and so on during disasters while referring to road network conditions as expressed using space syntax theory and hazardous elements visualized on the online map (Fig. 5).

## Experiment

As an experimental verification, we asked participants in groups of three to experience the process of making a disaster prevention map through a series of procedures (Table 1), then verified the utility of the proposed support.

**Fig. 6:**
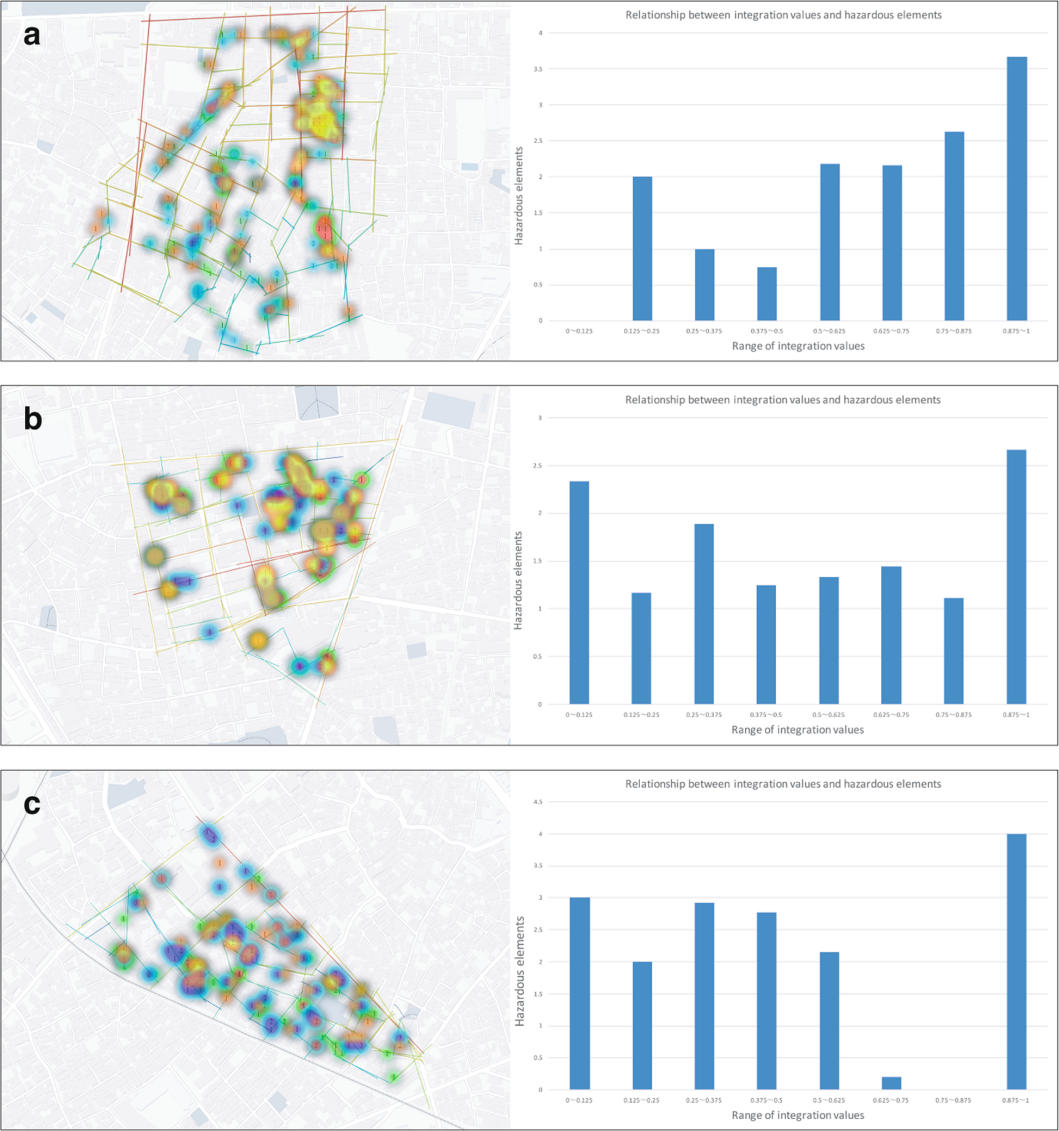
Relationship between road network and hazardous elements. **a)** Machiya-4chome **b)** Senju-Yanagicho **c)** Kyoujima-2chome

When performing experimental verifications, we referenced the Regional Earthquake Disaster Risk Measurement Survey (8th Edition) [1] published by the Tokyo Metropolitan Government, and selected the regions ranked 1st (Arakawa Ward, Machiya 4 area), 2nd (Arakawa Ward, Senju-yanagicho area), and 7th (Sumida Ward, Kyojima 2 area). The verifications in these three areas were performed with the cooperation of nine participants, who were assigned the task of collecting and evaluating hazardous elements using the abovementioned device (Fig. 3) while walking for about an hour through the target area. After that, we asked participants to simulate an effective disaster-time evacuation route on a tablet while observing the situation as visualized on the online map, thereby completing a disaster prevention map for each region. When simulating the evacuation route, we also distributed materials (Fig. 6) that graphed the relations between road network conditions and hazardous elements in each region analyzed after the fieldwork, using these as supplementary materials for selecting low-risk roads.

## Analysis and results

### Relationship between integration values and hazardous elements

In each experimental region, we analyzed the relations between road network conditions and hazardous elements collected by three participants through the fieldwork. First, in each region we counted the number of hazardous elements along each road while referring to the online map. Next, we normalized the Int.V value for each road as calculated using space syntax theory so that the minimum value was 0 and the maximum value was 1. As a result of aggregating and graphing the number of hazardous elements for the Int.V value of each road, we were able to clarify the characteristics of disaster risk in each region as shown in Fig. 6.

The Machiya 4-chome area is large and has an intricate road network, so we expected to find many hazardous elements on roads with low Int.V, but the analysis results showed a tendency for more hazardous elements to exist along roads near main streets with heavy traffic (roads with high Int.V) (Fig. 6a). We predicted that because the Senju-yanagicho area is well-developed and has good road connections, its hazardous elements would tend to be distributed throughout the area, and the analysis results supported that prediction (Fig. 6b). The Kyoshima 2-chome area has many narrow roads and intricate road networks, so we expected that many hazardous elements would be distributed throughout, but the analysis results showed that they were distributed mainly along roads with low Int.V (Fig. 6c).

In considering the relation between hazardous elements and road networks, we expected that roads with low Int.V would have many hazardous elements, but we learned that in reality there was a tendency for disaster risks to vary by region.

### Drawing evacuation plan

In Phase 3 of the process (Table 1), we asked participants to set their current location (start) and destination (goal), assuming a disaster had occurred. Participants also added evacuation shelters and other destinations as necessary, marking those locations on the online map.

**Fig. 7:**
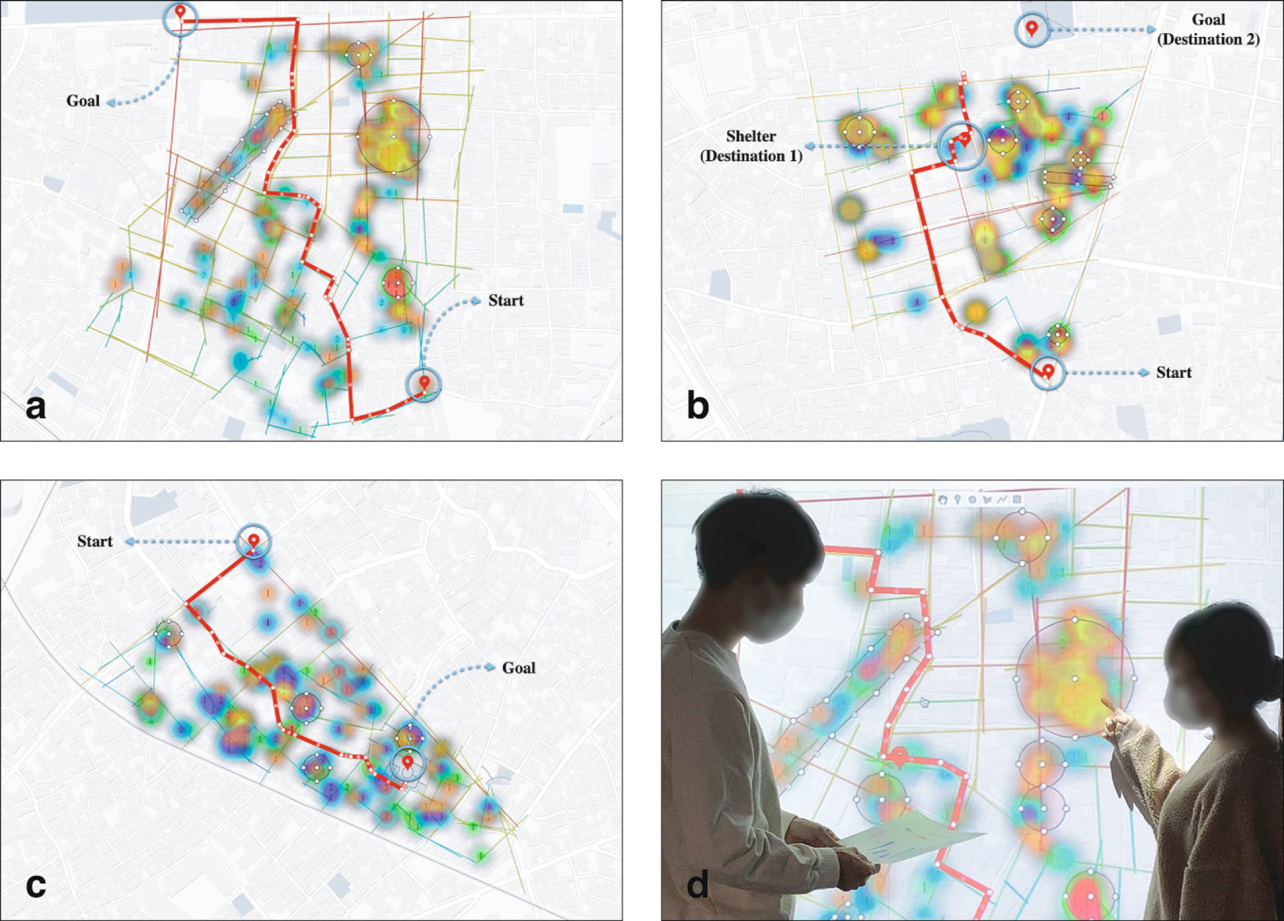
Drawing evacuation plan. **a)** Machiya-4chome **b)** Senju-Yanagicho **c)** Kyoujima-2chome **d)** Group discussion

Next, we asked them to report dangerous areas where hazardous elements are concentrated and to add a designator for risk aversion. Finally, referring to the materials (Fig. 6) showing graphs of the relation between road network conditions and hazardous elements, we asked them to plot an effective evacuation route on the online map, and to complete the disaster prevention map through discussions in groups of three (Fig. 7). In subsequent interviews, participants favorably reviewed the ability to intuitively simulate evacuation routes for risk aversion based on information visualized on the online map. We also heard opinions that these operations effectively reconfirmed regional risks.

### Support system for disaster prevention map making

The support system for creating disaster prevention maps developed in this research includes interactive operations for collecting and evaluating hazardous elements by using devices (Fig. 3) and simulating evacuation routes (Fig. 5). Therefore, after completing the disaster prevention map through a series of processes (Table 1), we conducted a usability test involving nine participants so that they could evaluate the disaster prevention map creation using this system. In the usability testing, we asked the participants to evaluate 10 questions by using a system usability scale (SUS) with five levels, from “I completely disagree” (1) to “I completely agree” (5) (Table 2).

**Table 2:** Results of usability evaluation using SUS

		Strongly				Strongly
		disagree				agree
		1	2	3	4	5
(1)	I often want to use this system.	0(0%)	0(0%)	5(55.6%)	4(44.4%)	0(0%)
(2)	I found this system unnecessarily complex.	1(11.1%)	5(55.6%)	3(33.3%)	0(0%)	0(0%)
(3)	I thought this system was easy to use.	0(0%)	0(0%)	6(66.7%)	2(22.2%)	1(11.1%)
(4)	You may need technical support to use this system.	0(0%)	3(33.3%)	1(11.1%)	5(55.5%)	0(0%)
(5)	felt that this system was simple and easy to operate.	0(0%)	2(22.2%)	0(0%)	5(55.6%)	2(22.2%)
(6)	This system was often inconsistent.	3(33.3%)	6(66.7%)	0(0%)	0(0%)	0(0%)
(7)	I think most people can learn how to use this system very quickly.	0(0%)	0(0%)	2(22.2%)	5(55.6%)	2(22.2%)
(8)	I found this system very awkward.	5(55.5%)	2(22.2%)	2(22.2%)	0(0%)	0(0%)
(9)	I am confident in using this system.	0(0%)	0(0%)	0(0%)	8(88.9%)	1(11.1%)
(10)	I thought I needed to learn a lot before I started using this system.	1(11.1%)	4(44.4%)	2(22.2%)	2(22.2%)	0(0%)

We next calculated the evaluation value for each item according to the following procedure. We subtracted 1 from responses to odd-numbered evaluation items, and we subtracted the values of responses to even-numbered evaluation items from 5. Finally, we multiplied the sum of both by 2.5 to calculate a value between 0 and 100 points. The mean result for evaluation values by the nine participants was 87.4 points (Table 3).

**Table 3:** SUS score

	Score
User 1	92.5
User 2	82.5
User 3	80.0
User 4	77.5
User 5	97.5
User 6	82.5
User 7	82.5
User 8	77.5
User 9	87.5
Average	87.4

We were thus able to obtain high scores as evaluation values from participants who used the proposed system to create disaster prevention maps. However, this score alone is insufficient for judging usability, so after completing the disaster prevention map creation task, we interviewed participants about their impressions of the disaster prevention map creation process and compiled the results.

The participants provided many positive opinions, such as the following: “The experience of using the device to collect hazardous elements gave me an opportunity to see roads with high risk in the region and increased my awareness of disaster prevention.” “The representation on the online map, which visualized the relation between hazardous elements and road networks, provided useful information for easily identifying roads with high risk in the region.” “I felt that it was good to be able to intuitively simulate disaster evacuation routes while looking at the disaster prevention information on the online map.” “I felt that it could be used as a pre-disaster prevention tool.”

We also received some negative comments, such as the following: “When a group walks the same route, the results could be biased and therefore prevent the correct evaluation of hazardous elements.” “The criteria for evaluating hazardous elements differ from person to person, so I felt that the guidelines should include more specific criteria.”

## Conclusion

In this research, we investigated the relations between road network conditions and hazardous elements in order to develop a support system for creating disaster prevention maps that visualize roads with high evacuation risk during a disaster, as well as simulate evacuation routes. To verify the usability of the system as a disaster prevention measure tool, we performed a series of processes (Table 1) through which experimental verifications yielded the following results in each phase.

In Phase 1, we confirmed that the experience of collecting hazardous elements via interaction with the device contributed to increasing awareness of disaster prevention among the participants. In Phase 2, we were able to visualize the characteristics of regional disaster risks by analyzing the relation between disaster factors and road network conditions (Fig. 6). In Phase 3, participants favorably reviewed the ability to intuitively simulate disaster evacuation routes while referring to online maps. In addition, we conducted usability testing regarding disaster prevention map creation using the proposed system, for which participants gave high evaluations (SUS scores) because it does not involve complicated operations (Tables 2 and 3). These results indicate that, as compared with existing disaster prevention map creation systems using WebGIS, user burden related to recording disaster prevention information on maps is reduced, and that the system effectively supports quicker disaster prevention measures and evacuation training.

In future studies, we hope to continue the practice of creating disaster prevention maps with this system, targeting areas with a high degree of regional risk, as designated by the Tokyo Metropolitan Government. We believe that by using this system to create disaster prevention maps in various regions and comparing road network conditions and hazardous elements data for each region, it would be possible to develop regional models indicating vulnerability or resilience to disasters. Doing so can be expected to provide useful materials for regional disaster prevention. Further improvement of this system will be critical to accomplishing this goal. In particular, developing applications for automatization of some of the manual tasks in disaster prevention map creation (e.g., calculations of Int.V and representation of road network conditions on the online map) is an urgent task. While addressing these issues, we hope to continue this research toward the ultimate realization of disaster prevention maps for local residents.

## Data Availability

All data generated or analyzed during this study are included in this article.

## References

[1] Bureau of Urban Development, Tokyo Metoropolitan Government (2018) Regional risk measurement survey report on earthquake. 8th. Tokyo Metoropolitan Government. https://www.toshiseibi.metro.tokyo.lg.jp/bosai/chousa_6/home.htm. Accessed 29 July 2021.

[2] Kanai M, Arikawa K, Katada T (2017). The study on standard of actualreference and keeping states conditions of the hazard map of residents. J Dis Informat Stud.

[3] Ushiyama M, Yoshida A, Kashiwagi N, Sato S, Sato Y (2009). Analysis of the effects of disaster workshop for non-residents on the participant. J JSNDS.

[4] Enokida S, Fukushima T, Yoshino T, Sugimoto K, Egusa N (2017) Proposal of Town-walk type disaster-preparedness map making support system. Paper presented at the multimedia, distributed, cooperative, and mobile symposium, Executive Committee Member, 28-30 June 2017.

[5] Tajima S, Murakami Y, Uchida O, Kajita Y (2018). Evaluating Lectures Concerning Information Ethics in Teachers College: LearnerŠs Confidence toward Creating Lesson Plans. J Japan Soc Educat Technol.

[6] Murakoshi T, Yamamoto K (2014). Study on a social media GIS to support the utilization of disaster information: for disaster reduction measures from normal times to disaster outbreak times. J Socio-Informat.

[7] Kubota S, Soga K, Sasaki Y, Miura T, Takisawa H, Sasaki T (2012). Development and operational evaluation of regional social networking service as public participation GIS. Theory Appl GIS.

[8] Hiller B, Leaman A, Stansall P, Bedford M (1976). Space syntax. Environ Plann B.

[9] Takahashi R, Hirano K (2010). Street network characteristics and green space recognition. Lect Landsc Des Stud.

[10] Nagaie T, Hokao K, Inohae T (2008). Analysis of the relationships between the accesibility of urban space based on the Space Syntax Theory and the opportunity crime, the police’s perceived risk of crime. J City Plann Inst Jpn.

[11] DepthmapX. https://www.ucl.ac.uk/bartlett/architecture/research/space-syntax/depthmapx. Accessed 29 July 2021.

[12] Turner A (2001) Depthmap: a program to perform visibility graph analysis In: Abstracts of the 3rd international symposium on space syntax, Georgia Institute of Technology, Atlanta, 7–11 May 2001.

[13] Ministry of Land, Infrastructure. https://www.mlit.go.jp/policy/shingikai/content/001354001.pdf. Accessed 2 Apr 2021.

